# STAT1 epigenetically regulates LCP2 and TNFAIP2 by recruiting EP300 to contribute to the pathogenesis of inflammatory bowel disease

**DOI:** 10.1186/s13148-021-01101-w

**Published:** 2021-06-10

**Authors:** Ya-Li Yu, Meng Chen, Hua Zhu, Ming-Xing Zhuo, Ping Chen, Yu-Juan Mao, Lian-Yun Li, Qiu Zhao, Min Wu, Mei Ye

**Affiliations:** 1 Department of Gastroenterology, Zhongnan Hospital, Wuhan University, Wuhan, 430071 Hubei China; 2 Hubei Clinical Centre and Key Laboratory of Intestinal and Colorectal Diseases, Zhongnan Hospital, Wuhan University, Wuhan, 430071 Hubei China; 3 Frontier Science Center for Immunology and Metabolism, Hubei Key Laboratory of Cell Homeostasis, Hubei Key Laboratory of Developmentally Originated Disease, Hubei Key Laboratory of Intestinal and Colorectal Diseases, College of Life Sciences, Wuhan University, Wuhan, 430072 Hubei China

**Keywords:** IBD, Enhancer, H3K27ac, STAT1, EP300

## Abstract

**Background:**

The aetiology of inflammatory bowel disease (IBD) is related to genetics and epigenetics. Epigenetic regulation of the pathogenesis of IBD has not been well defined. Here, we investigated the role of H3K27ac events in the pathogenesis of IBD. Based on previous ChIP-seq and RNA-seq assays, we studied signal transducer and activator of transcription 1 (STAT1) as a transcription factor (TF) and investigated whether the STAT1–EP300–H3K27ac axis contributes to the development of IBD. We performed ChIP-PCR to investigate the interaction between STAT1 and H3K27ac, and co-IP assays were performed to investigate the crosstalk between STAT1 and EP300.

**Results:**

Lymphocyte cytosolic protein 2 (LCP2) and TNF-α‐inducible protein 2 (TNFAIP2) are target genes of STAT1. p-STAT1 binds to the enhancer loci of the two genes where H3K27ac is enriched, and EP300 subsequently binds to regulate their expression. In mice with dextran sulfate sodium (DSS)-induced acute colitis, an EP300 inhibitor significantly inhibited colitis.

**Conclusions:**

p-STAT1 and EP300 promote TNFAIP2 and LCP2 expression through an increase in H3K27ac enrichment on their enhancers and contribute to the pathogenesis of chronic inflammation.

**Graphic abstract:**

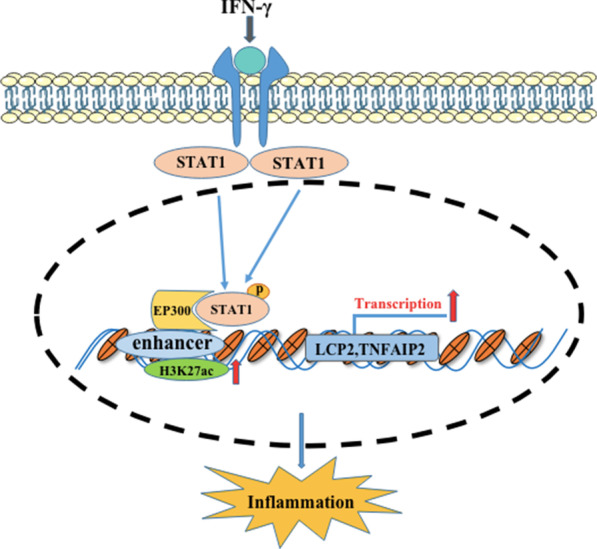

## Introduction

Inflammatory bowel disease (IBD) is a nonspecific chronic inflammatory disorder that occurs in the intestinal tract and is caused by multiple factors. Two types of IBD have been identified: ulcerative colitis (UC) and Crohn’s disease (CD). IBD is related to factors such as genetics, the environment, immunity, and intestinal microbiota [1]. Environmental factors have been shown to increase the risk of disease development by changing epigenetic patterns [2, 3]. For example, smoking changes the epigenetic pattern of airway cells in humans and affects genes expression [4]. Particulate matter in air and airborne benzene induce inflammation and carcinogenesis by changing the pattern of epigenetic modifications [5, 6]. In recent years, the role of epigenetic mechanisms in IBD has attracted increasing attention.

Epigenetic modifications are biochemical changes in chromatin that do not affect the nucleotide sequence of the genome. These modifications explain the shaping of the immune system throughout the lifetime, as well as the effects of external environmental factors, microbes, and nonmicrobial particles during the development of diseases. Epigenetic modifications include DNA methylation, histone modifications, and noncoding RNAs [7]. DNA methylation affects the duration and severity of IBD, the extent of inflammation, the hospitalization rate, and the possibility of canceration [8]. The DNA methylation pattern in the intestinal epithelial cells (IECs) of children with CD was significantly different from that in the normal group and was closely related to the prognosis of the CD [9]. Tahara et al. [10] showed that a large number of hypermethylated sites are located in the CpG islands in the rectal inflammatory mucosa of patients with UC, and methylation in these patients is closely related to the disease course. Noncoding RNAs regulate gene expression at both the transcriptional and posttranscriptional levels and participate in the onset and progression of IBD by modifying T-cell differentiation, IL-23/Th17 signaling pathways, and autophagy [11]. The role of histone modification in IBD is also important. Histone modification refers to the process of covalent modifications on histones, such as methylation, acetylation, phosphorylation, and adenylation. Inhibition of histone deacetylases (HDACs) proved that histone 3 acetylation is related to dextran sulfate sodium (DSS)-induced colitis in mice [12], and acetylation of histone 4 in the colonic mucosa was significantly increased in mice with TNBS-induced colitis [13]. Furthermore, the E3 ligase FBXW7 promotes the expression of Ccl2 and Ccl7 by suppressing H3K27me3 modification via degradation of the histone–lysine N-methyltransferase EZH2 in macrophages, thereby promoting the aggregation of proinflammatory mononuclear phagocytes in local colonic tissues [14]. Li et al. [15] proved that H3 acetylation was significantly reduced in the colonic epithelium of individuals with UC and negatively correlated with the disease severity. In addition, H3K27ac plays a role in specific genomic regions in mice with DSS-induced colitis [16]. ChIP-seq showed increased H3K27ac levels at the promoters of iNOS, IL-6, TNF-α, and other inflammatory factors in the colon tissues of mice exposed to azoxymethane (AOM)/DSS [8]. EP300 is a transcriptional cofactor with acetyltransferase activity that promotes the acetylation of histone and nonhistone proteins [17]. EP300 acetylates H3K27 and contributes to inflammation, differentiation, lipid metabolism, and chromatin remodeling [18, 19]. In the experimental Drosophila embryonic mesoderm and mouse models, H3K27ac enrichment is closely related to promoter and enhancer activity regulated by EP300 [19–23]. CBP/EP300 suppresses immunity by promoting the acetylation of H3K27 on enhancers and promoters of tumor-promoting target genes in myeloid-derived suppressor cells (MDSCs) to promote tumorigenesis [24]. Increased expression of EP300 induces H3K27 acetylation and leads to higher concentrations of lithocholic acid (LCA) and deoxycholic acid (DCA) to promote colon cancer [25]. Currently, the effect of EP300-H3K27ac on inflammation remains elusive.

In a previous study, by applying a high-throughput ChIP-seq assay for H3K27ac and an RNA-seq assay in a mouse model of DSS-induced chronic colitis, we revealed changes in the global genomic profile and investigated its role in the pathogenesis of IBD, indicating that H3K27ac levels were increased on enhancers in colon tissues from the DSS group and were involved in DSS-induced colitis [26]. In the current study, based on a previous ChIP-seq assay, we studied the transcription factor (TF) signal transducer and activator of transcription 1 (STAT1) and investigated whether the STAT1–EP300–H3K27ac axis contributes to the development of IBD.

## Results

### Increased levels of STAT1/p-STAT1 in a mouse model of DSS-induced chronic colitis and in patients with IBD are related to H3K27ac modification

**Fig. 1:**
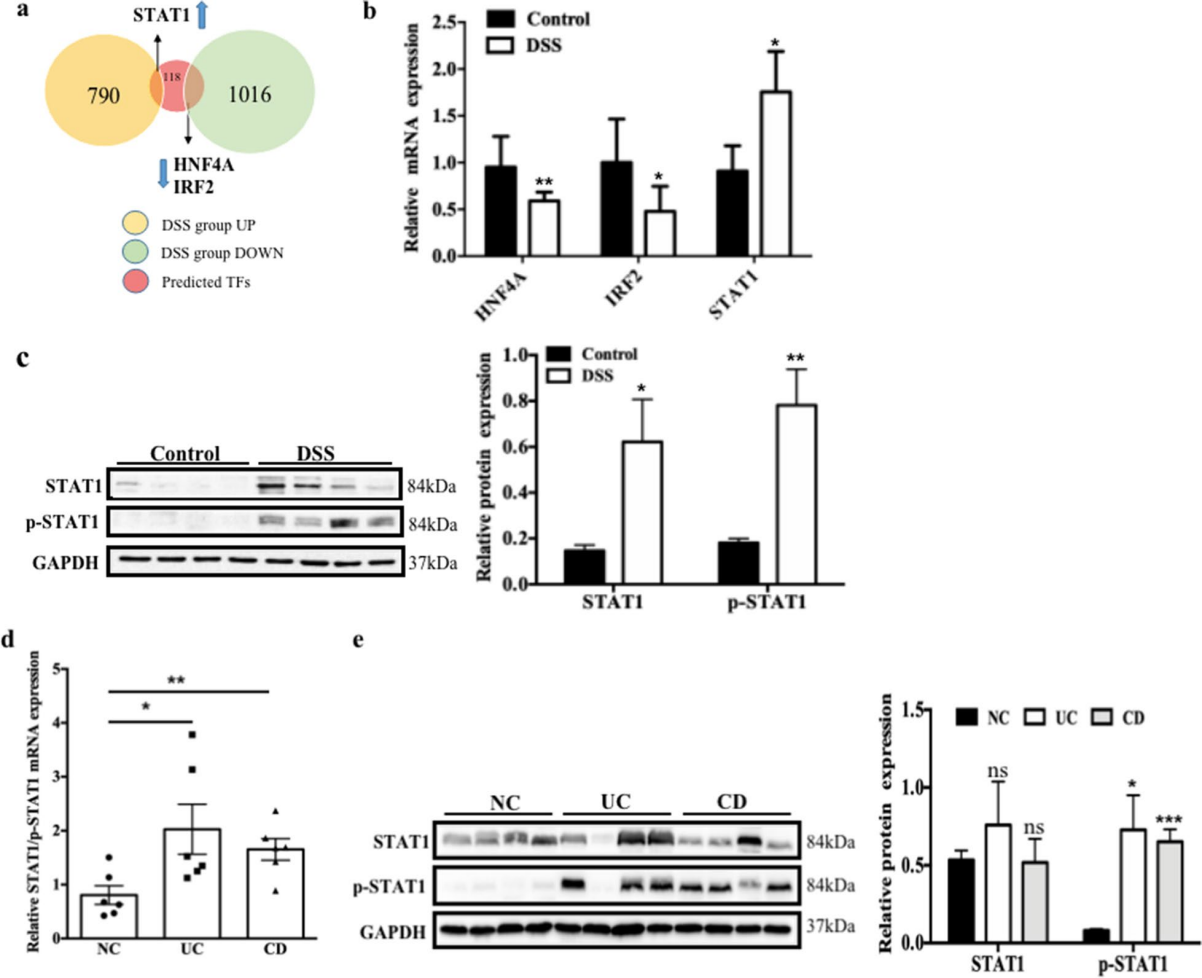
Increased levels of STAT1/p-STAT1 in a mouse model of DSS-induced chronic colitis and in patients with IBD are related to H3K27ac modification. **a** Overlap of the predicted TFs with the differentially expressed genes by RNA-seq in DSS-induced mice chronic colitis. **b** STAT1, HNF4A, and IRF2 mRNA expression in DSS-induced mice chronic colitis (error bars: mean ± SEM; control group *n* = 7, DSS group *n* = 9). **c** STAT1/p-STAT1 protein expression in DSS-induced mice chronic colitis. **d-e** STAT1/p-STAT1 mRNA and protein expression in IBD patients (error bars: mean ± SEM; NC group *n* = 8, UC group *n* = 8, CD group *n* = 10). *p < 0.05, **p < 0.01, ***p < 0.001, ns means no significance

Based on the H3K27ac ChIP-seq assay, we predicted 118 TFs that bind to the enhancers where H3K27ac was enriched in inflamed intestinal tissues from mice with DSS-induced chronic colitis, and the TFs were then overlapped with differentially expressed genes (DEGs) identified in our previous RNA-seq analysis [26]. We identified the three most significantly differentially expressed TFs, among which STAT1 was upregulated and HNF4A and IRF2 were downregulated (Fig. 1a). We further verified the expression of these TFs in mice with chronic colitis, and the result was consistent with our prediction (Fig. 1b). Because the H3K27ac modification is usually associated with increased gene expression, we selected a functionally upregulated TF, STAT1, for further study. Levels of the STAT1 and p-STAT1 proteins were increased after DSS treatment (Fig. 1c), and the level of the STAT1/p-STAT1 mRNA in the inflamed mucosa of patients with IBD was increased compared to that in the normal mucosa (Fig. 1d). However, the level of the p-STAT1 protein, but not total STAT1 protein, was increased (Fig. 1e). These results suggested the involvement of the TF STAT1 in IBD might be related to H3K27ac modification.

### Identification of lymphocyte cytosolic protein 2 (LCP2) and TNF-α-inducible protein 2 (TNFAIP2) as STAT1 target genes

**Fig. 2:**
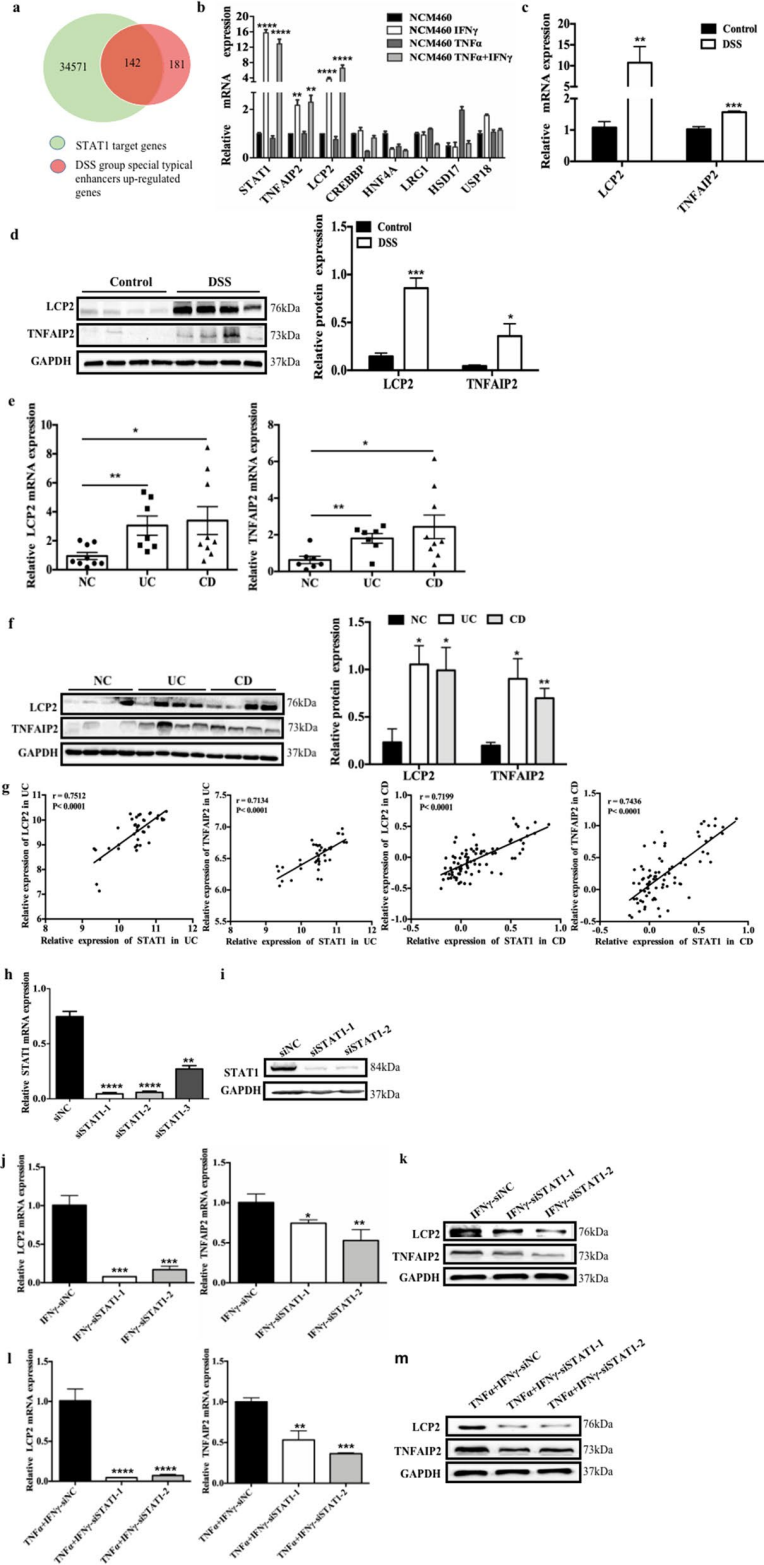
Identification of *LCP2* and *TNFAIP2* as STAT1 target genes.** a** Overlap of the target of STAT1 on the website of GTRD and the upregulated genes in the RNA-seq whose enhancers had increased H3K27ac enrichment after DSS treatment. **b** TNFAIP2, LCP2, CREBBP, HNF4A, LRG1, HSD17, USP18 mRNA expression in NCM460 cell inflammation model (error bars: mean ± SD). **c-d** LCP2 and TNFAIP2 mRNA and protein expression in DSS-induced mice chronic colitis (error bars: mean ± SEM; control group *n* = 7, DSS group *n* = 9). **e–f** LCP2 and TNFAIP2 mRNA and protein expression in IBD patients (error bars: mean ± SEM; NC group *n* = 8, UC group *n* = 8, CD group *n* = 10). **g** The correlation between STAT1 and LCP2, TNFAIP2 in UC and CD patients in GEO database**. h–i** STAT1 mRNA and protein expression in NCM460 after transfected with siSTAT1-1, 2, 3 or the negative control (error bars: mean ± SD). **j–k** LCP2 and TNFAIP2 mRNA and protein expression in NCM460 after transfected with siSTAT1-1, 2 or the negative control with stimulation by IFN-γ (error bars: mean ± SD). **l–m** LCP2 and TNFAIP2 mRNA and protein expression in NCM460 after transfected with siSTAT1-1, 2 or the negative control with stimulation by TNF-α and IFN-γ (error bars: mean ± SD). *p < 0.05, **p < 0.01, ***p < 0.001, ****p < 0.0001

We sought to identify the target genes of STAT1 that were upregulated in mice with DSS-induced colitis and to further investigate the mechanism underlying the contribution of STAT1 to IBD through H3K27ac. Based on previous ChIP-seq and RNA-seq assays, we identified 181 upregulated genes whose enhancers had increased H3K27ac enrichment after the DSS treatment [26]. After overlapping the 181 genes with the STAT1 target genes in the Gene Transcription Regulation Database (GTRD, http://gtrd.biouml.org), we obtained 142 genes as candidate target genes of STAT1 (Fig. 2a), and the top seven candidate genes with the most significant changes (TNFAIP2, LCP2, CREBBP, HNF4A, LRG1, HSD17, and USP18) were selected for verification. We built an inflammatory cell model by stimulating NCM460 cells with TNF-α and/or IFN-γ and found that STAT1 was activated only when IFN-γ was present, consistent with the results from previous reports [27–29]. Moreover, we confirmed that LCP2 and TNFAIP2 were upregulated, consistent with the sequencing results (Fig. 2b). In addition, both genes were upregulated in the mice with DSS-induced colitis and patients with IBD (Fig. 2c–f). We analyzed the data in the GEO (Gene Expression Omnibus) database and found that STAT1 expression was positively correlated with the expression of LCP2 and TNFAIP2 in patients with UC (GES107499-GPL15207) and CD (GSE20881-GPL1708-20418) (Fig. 2g). Furthermore, LCP2 and TNFAIP2 were stably downregulated in cells transfected with the STAT1 siRNA (Fig. 2h–i) upon IFN-γ (Fig. 2j–k) or TNF-α and IFN-γ cotreatment (Fig. 2l–m), indicating that LCP2 and TNFAIP2 are target genes of STAT1. Taken together, these results showed that LCP2 and TNFAIP2 were target genes of STAT1.

### STAT1 regulates its target genes through H3K27ac at their enhancers in mice with DSS-induced chronic colitis

**Fig. 3:**
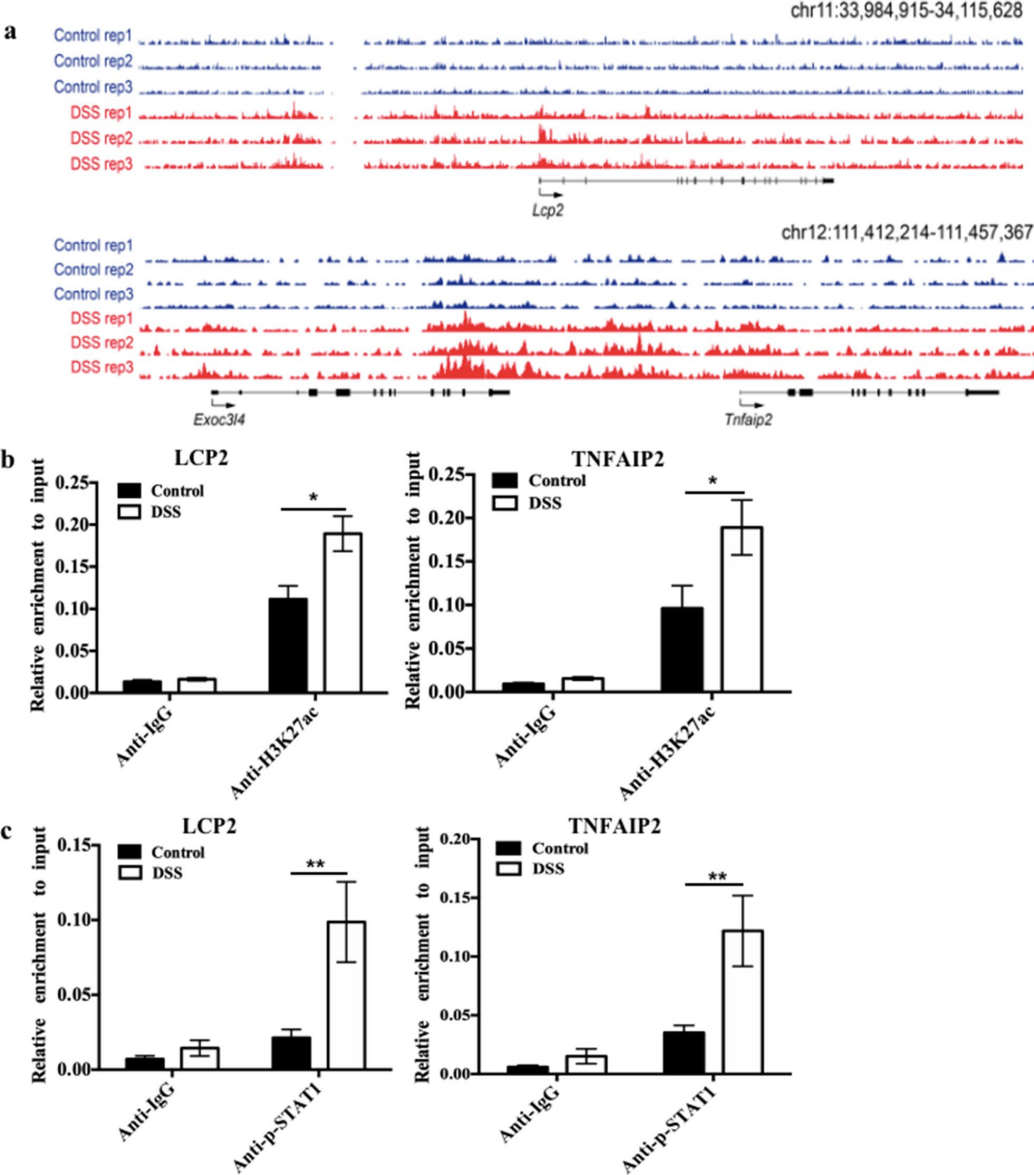
STAT1 regulates its target genes through H3K27ac at their enhancers in mice with DSS-induced chronic colitis.** a** UCSC browser view showing the ChIP-seq density of H3K27ac signal in both control tissue and DSS-induced tissue located in LCP2 and TNFAIP2 gene locus. **b** ChIP-PCR was performed to validate H3K27ac modification at the LCP2 and TNFAIP2 enhancer in DSS-induced mice chronic colitis. **c** ChIP-PCR was performed to validate the occupancy of p-STAT1 on the LCP2 and TNFAIP2 enhancer where H3K27ac enriched in DSS-induced mice chronic colitis. Error bars: mean ± SEM; *n* = 6 per group. *p < 0.05, **p < 0.01

We confirmed that LCP2 and TNFAIP2 were the target genes of STAT1 and that H3K27 acetylation was enriched at their enhancers according to previous ChIP-seq results [26] (Fig. 3a). We verified by ChIP-PCR that H3K27ac enrichment was increased at the enhancers of LCP2 and TNFAIP2 in mice with DSS-induced colitis (Fig. 3b). These results implied a potential role for H3K27ac enrichment at enhancers in promoting inflammation.

Next, we sought to investigate whether STAT1 modulates target gene expression through H3K27ac at enhancers. By performing ChIP-PCR experiments, we found that the recruitment of p-STAT1 to the enhancers in the LCP2 and TNFAIP2 promoters was significantly increased in tissues of mice with chronic colitis compared to mice in the control group (Fig. 3c). Therefore, p-STAT1 may promote the expression of LCP2 and TNFAIP2 by increasing H3K27ac enrichment at the enhancers of these two genes.

### p-STAT1 may recruit EP300 to promote target gene expression in NCM460 cells

**Fig. 4:**
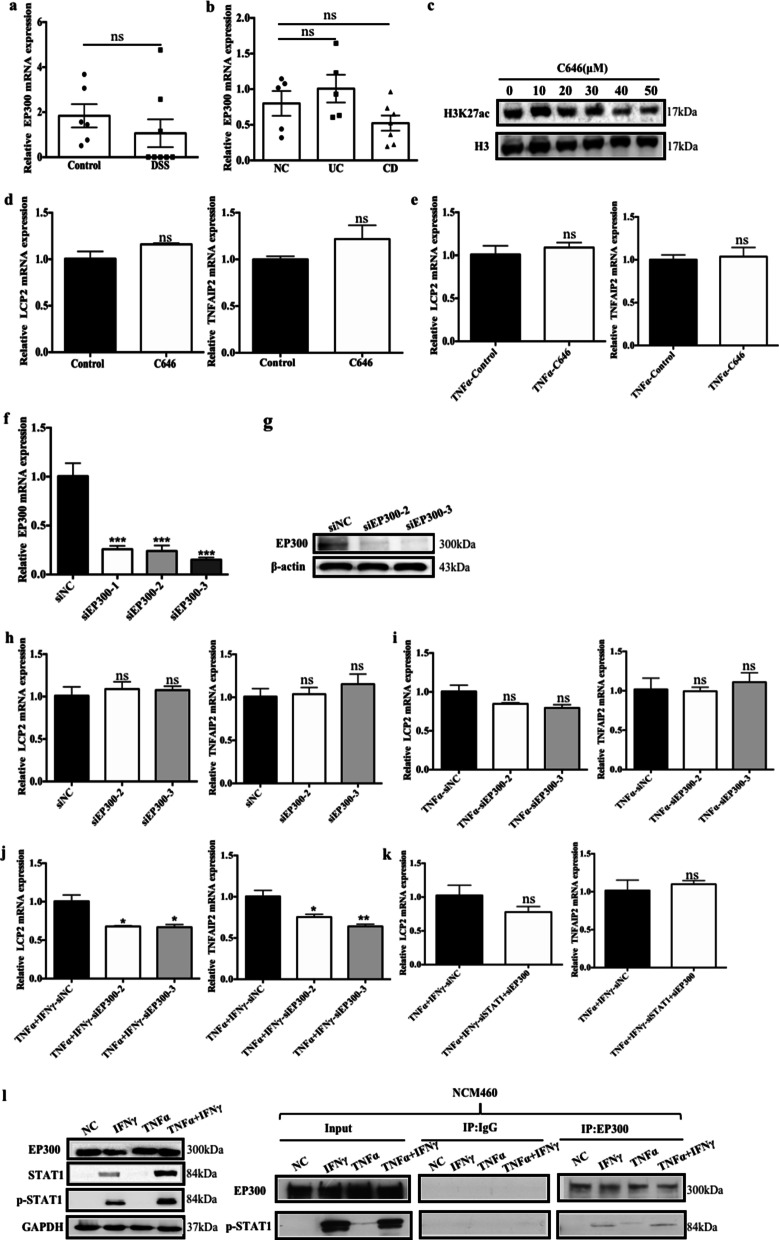
p-STAT1 may recruit EP300 to promote target gene expression in NCM460 cells. **a** EP300 mRNA expression in DSS-induced mice chronic colitis (error bars: mean ± SEM; control group *n* = 7, DSS group *n* = 9). **b** EP300 mRNA expression in IBD patients (error bars: mean ± SEM; NC group *n* = 8, UC group *n* = 8, CD group *n* = 10). **c** H3K27ac protein expression in NCM460 after adding EP300 inhibitor C646 in different concentration (0, 10, 20, 30, 40, 50 μM) for 24 h. **d–e** LCP2 and TNFAIP2 mRNA expression in NCM460 after adding C646 or DMSO without or with stimulation of by TNF-α (error bars: mean ± SD). **f–g** EP300 mRNA and protein expression in NCM460 after transfected with siEP300-1, 2, 3 or the negative control (error bars: mean ± SD). **h–i** LCP2 and TNFAIP2 mRNA expression in NCM460 after transfected with siEP300 or the negative control with or without stimulation by TNF-α (error bars: mean ± SD). **j** LCP2 and TNFAIP2 mRNA expression in NCM460 after transfected with siEP300 or the negative control with stimulation by TNF-α + IFN-γ (error bars: mean ± SD). **k** LCP2 and TNFAIP2 mRNA expression in NCM460 after transfected with siSTAT1 + siEP300 or the negative control with stimulation by TNF-α + IFN-γ (error bars: mean ± SD). **l** co-IP experiment was performed to validate the combination between p-STAT1 and EP300. *p < 0.05, **p < 0.01, ***p < 0.001, ns means no significance

Since H3K27ac is catalyzed mainly by the epigenetic enzyme EP300, we speculate that EP300 may play a role in the regulatory effect of STAT1 on LCP2 and TNFAIP2 expression. No significant differences in the expression of EP300 in the inflamed tissues from either mice with chronic colitis (Fig. 4a) or patients with IBD (Fig. 4b) were observed. First, we verified that the EP300 inhibitor C646 [30] was effective in NCM460 cells (Fig. 4c). In NCM460 cells with or without TNF-α induction, the expression of the LCP2 and TNFAIP2 mRNA did not show a significant change when EP300 was inhibited (Fig. 4d–e). Furthermore, we silenced EP300 with siRNAs and selected the most efficient sequence for further study (Fig. 4f–g). Consistent with the results obtained with the C646 inhibitor, the expression of the LCP2 and TNFAIP2 mRNAs did not show a significant change after silencing EP300 in NCM460 cells stimulated with or without TNF-α (Fig. 4h–i). When EP300 was silenced in NCM460 cells and STAT1 was activated by IFN-γ, the induction of LCP2 and TNFAIP2 expression was repressed (Fig. 4j). As shown in Fig. 2h–i, siSTAT1 decreased the expression of the two genes in the absence of EP300. However, after silencing STAT1 and EP300 simultaneously, the expression of the LCP2 and TNFAIP2 mRNAs did not change (Fig. 4k). These results indicated that EP300 is required for STAT1 to regulate TNFAIP2 and LCP2 expression. Moreover, the western blot analysis results showed that the levels of total STAT1 and p-STAT1 increased upon IFN-γ stimulation, and immunoprecipitation results indicated that p-STAT1 bound to EP300 upon IFN-γ treatment (Fig. 4l). Taken together, these results indicate that EP300 is required for STAT1 to induce LCP2 and TNFAIP2 expression.

### Inhibition of EP300 relieves DSS-induced colitis in mice

**Fig. 5:**
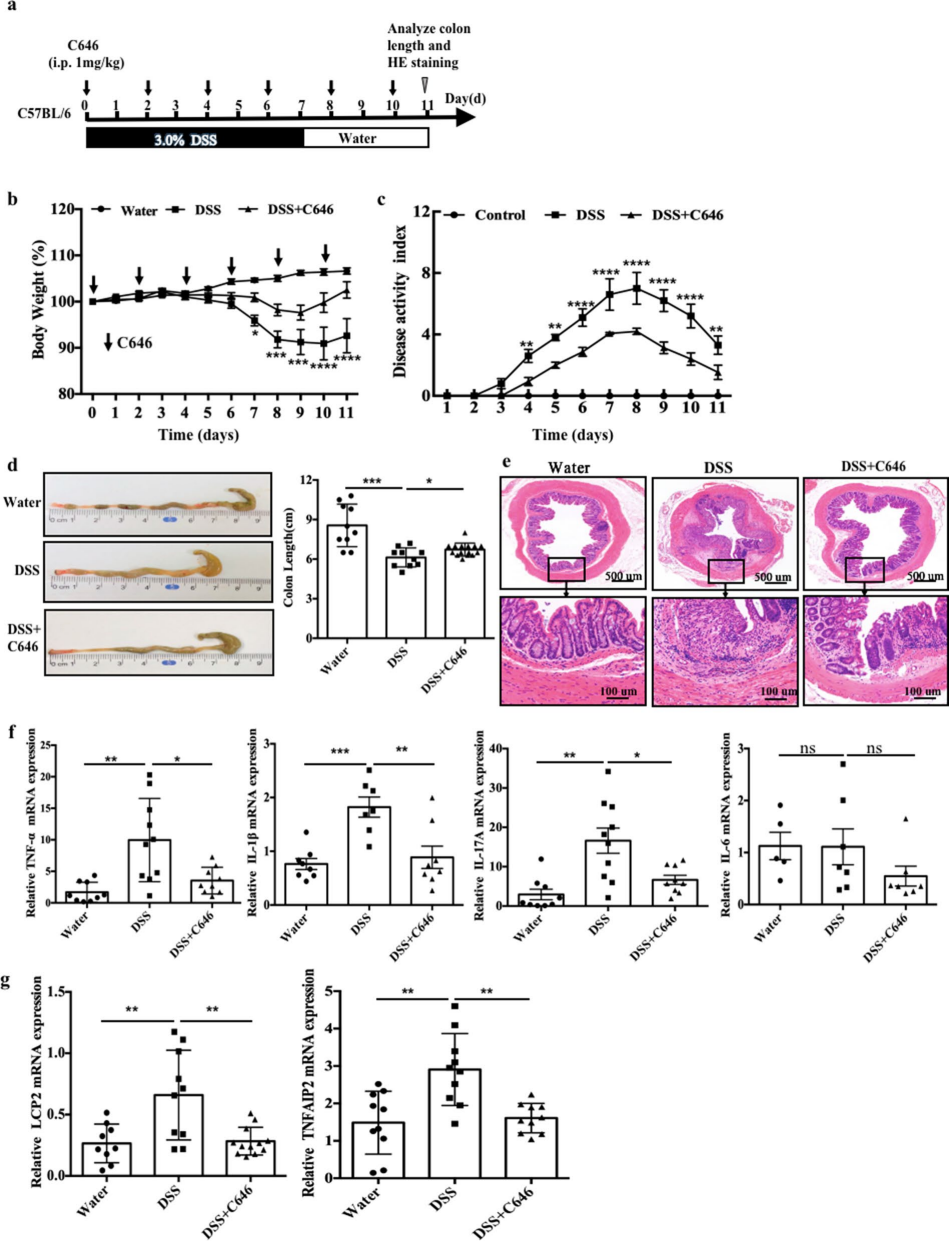
Inhibition of EP300 relieves DSS-induced colitis in mice. **a** Methods for DSS-induced acute colitis in C57BL/6 mice and C646 administration. **b** Body weight and **c** disease activity index of mice that received regular drinking water alone (water group *n* = 10) or 3.0% DSS-containing water (DSS group *n* = 10), or 3.0% DSS combined with C646 injection (DSS + C646 group *n* = 15). For statistical comparisons, asterisk indicates DSS vs. DSS + C646. **d** Colon length and **e** representative hematoxylin and eosin (H&E) staining of distal colon sections at day 11 after DSS induction (water group and DSS group *n* = 10, DSS + C646 group *n* = 15). **f** Colonic inflammatory cytokine analysis from colonic tissue at day 11 after DSS induction (*n* = 10 per group). **g** LCP2 and TNFAIP2 mRNA expression in colonic tissue at day 11 after DSS induction (*n* = 10 per group). Error bars: mean ± SEM. *p < 0.05, **p < 0.01, ***p < 0.001, ****p < 0.0001, ns means no significance

We found that p-STAT1 binds to EP300 to regulate the expression of LCP2 and TNFAIP2 by promoting H3K27 acetylation at enhancers, and thus, we further investigated whether the increase in H3K27ac levels mediated by EP300 is involved in the development of colitis in mice. We established a mouse model of DSS-induced acute colitis as previously reported [31]. DMSO (control) or C646 was intraperitoneally (i.p.) injected at different time points during the course of colitis development (Fig. 5a). After the administration of C646, the weight loss of mice treated with DSS was significantly alleviated (Fig. 5b). The disease activity index (DAI), a score used to evaluate the clinical manifestations of colitis, was also decreased upon C646 treatment (Fig. 5c). In addition, the extent of colon shortening is considered a macroscopic indicator reflecting the degree of intestinal inflammation. We observed significantly longer colons after DSS exposure in C646-treated mice than in mice in the DMSO-treated group (Fig. 5d). The detailed histological analysis of colonic lesions in colitis mice showed severe pathology, such as extensive destruction of crypt structures and abundant inflammatory cell infiltration. Our histological analysis of the mouse colon showed that these pathological changes were significantly reduced after the administration of C646, as the mucosal structure was well preserved and the infiltration of inflammatory cells and the loss of crypts were significantly reduced (Fig. 5e). Proinflammatory factors are closely related to intestinal inflammation and clinical symptoms of IBD [32]. We next explored whether C646 treatment affected the production of proinflammatory factors. We detected the expression of proinflammatory factors, including TNF-α, IL-1β, IL-6, and IL-17A, in mouse colon tissues and found that C646 administration led to a significant decrease in the levels of TNF-α, IL-1β, and IL-17A, whereas the level of IL-6 remained unaffected (Fig. 5f). Furthermore, the mRNA expression of LCP2 and TNFAIP2 was upregulated in mice with DSS-induced colitis, and these changes were reversed after C646 administration (Fig. 5g). Therefore, a decrease in H3K27 acetylation induced by inhibiting EP300 alleviates DSS-induced colitis in mice.

## Discussion

Epigenetic modifications have been extensively studied in various human diseases, and their effect on autoimmune diseases has drawn increasing attention. As the major event in epigenetic modification, some studies have shown that H3K27 acetylation plays a role in various cancers, such as increased H3K27ac signals in colon cancer [33] and hepatocellular carcinoma [34]. However, to date, the contribution of epigenetic events to the pathogenesis of IBD remains elusive. The results of our previous ChIP-seq assay indicated that H3K27ac levels at enhancers tended to increase during colitis, suggesting that this modification might be involved in intestinal inflammation. Thus, H3K27ac enrichment at enhancers might contribute to the pathogenesis of IBD.

The binding of TFs to enhancers is one of the critical steps in transcriptional activation. The deposition of H3K27ac unwinds chromatin and then promotes the regulation of target gene expression by TFs [35, 36]. According to a previous ChIP-seq assay, we identified STAT1 as an important TF involved in this process. STAT1 activity is increased in some inflammatory diseases, such as asthma and rheumatoid arthritis [37, 38]. When activated, the protein translocates into the nucleus and binds to specific promoter elements to regulate gene expression [39]. It directly binds to the sphingosine 1-phosphate receptor 1 (S1PR1) promoter at the region between bp -29 to bp -12 and promotes S1PR1 expression, which induces the development of cancers and inflammatory diseases [40]. The phosphorylation of STAT1 (Y701 and S727) is substantially increased upon DSS exposure, but a significant change in total STAT1 levels is not observed [41]. In patients with CD and UC, the levels and activity of STAT1 and p-STAT1 are also increased [39]. In our study, STAT1 and p-STAT1 levels were elevated in vivo and in vitro, consistent with previous reports. We also verified that the level of p-STAT1 but not STAT1 was increased in patients with IBD. Collectively, these data implicated STAT1 as a TF involved in regulating H3K27ac modification and contributing to the pathogenesis of IBD.

Then, we identified LCP2 and TNFAIP2 as the target genes of STAT1 by overlapping the target genes of STAT1 predicted by GTRD and our RNA-seq assay. The expression of the two genes was increased in activated NCM460 cells, the inflamed tissues from mice with DSS-induced chronic colitis and patients with IBD. We analyzed the data in the GEO database and found that STAT1 expression was positively correlated with the expression of LCP2 and TNFAIP2 in patients with IBD. In addition, silencing STAT1 in intestinal epithelial cells induced downregulation of both genes. TNFAIP2 is a proinflammatory gene whose expression is regulated by multiple TFs and signaling pathways, including the NF-κB, KLF5, and retinoic acid pathways, which play essential roles in inflammation [42]. LCP2 (SLP-76) is a direct regulator of nuclear pore function in T-cells and is a critical immune cell involved in the pathogenesis of IBD [43].

Furthermore, we verified that H3K27ac levels were increased at the enhancers of these two genes in mice with DSS-induced chronic colitis and confirmed the relationship of H3K27ac with the expression of TNFAIP2 and LCP2. Van der Kroef et al. reported that H3K27ac is enriched at the promoters of myxoma resistance protein 1 (MX1) and cytidine/uridine monophosphate kinase 2 (CMPK2) in SSc and that STAT1 strongly binds the hyperacetylated regions in SSc [44]. In the current study, the binding of p-STAT1 to the enhancers of TNFAIP2 and LCP2 was significantly increased in tissues from mice with chronic colitis, suggesting that both p-STAT1 deposition and H3K27ac enrichment occur on the same enhancer of both the TNFAIP2 and LCP2 genes.

EP300 is an acetylase that regulates H3K27 acetylation. A previous study showed that STAT1 requires EP300 for effective transcriptional activity [45], and STAT1 also interacts with p300/CBP through its C-terminal transactivation domains (TADs) [46]. EP300 and STAT1/3 act cooperatively to participate in the pathogenesis of light-induced retinopathy in zebrafish [47]. STAT1 activity is regulated by EP300-dependent acetylation, and that the interaction between EP300 and STAT1 participates in oxidized low-density lipoprotein uptake and foam cell formation, which are responsible for the pathogenesis of atherosclerosis [48, 49]. In the present study, STAT1 could not regulate target gene expression without EP300, and we verified that p-STAT1 interacted with EP300 to increase H3K27ac levels and upregulate TNFAIP2 and LCP2 expression. Moreover, we applied an inhibitor of EP300, C646, to suppress the activity of EP300 in vivo, and this treatment significantly alleviated colitis in mice. Therefore, STAT1 regulates TNFAIP2 and LCP2 by binding to EP300 to promote H3K27ac enrichment at their enhancers. Furthermore, strategies targeting EP300 might be a promising treatment for IBD.

## Conclusions

**Fig. 6:**
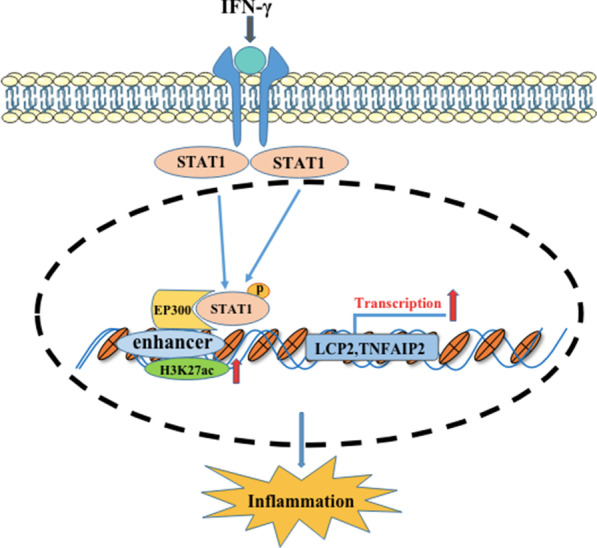
Model explaining how p-STAT1 interacts with EP300 to promote H3K27ac enrichment on the enhancers of LCP2 and TNFAIP2 and contributes to the development of chronic inflammation. When STAT1 is activated by IFN-γ, STAT1 migrates into the nucleus and binds to the enhancers of LCP2 and TNFAIP2. p-STAT1 interacts with EP300 to promote H3K27ac enrichment on the enhancers and promote the transcriptions of LCP2 and TNFAIP2 and then contributes to the development of chronic inflammation

Based on previous ChIP-seq and RNA-seq assays, we verified in the current study that p-STAT1 interacts with EP300 to promote H3K27ac enrichment at the enhancers of LCP2 and TNFAIP2 and contributes to the development of chronic inflammation (Fig. 6). Furthermore, H3K27 acetylation might be a promising therapeutic target for IBD.

## Materials and methods

### Specimen collection

A total of ten patients with clinically active Crohn’s disease (CD) and eight patients with active ulcerative colitis (UC) were collected in the study at Zhongnan Hospital of Wuhan University (Wuhan, China) between August 2018 and February 2019. All patients were diagnosed by endoscopic features, and original histopathological detection and a history of autoimmune diseases were excluded. None of the patients received steroids, immunosuppressives, or biologic agents. Normal controls (*n* = 8) were age- and sex-matched healthy volunteers. Inflamed tissues of UC and CD collected during endoscopic procedure or operation were preserved in liquid nitrogen. Clinical data of patients were also collected.

### Cell culture and treatment

Cell lines NCM460 was purchased from the China Center for Type Culture Collection (Wuhan, China), and cells had been authenticated for STR profiling and tested for mycoplasma by the vendor. All the cells were cultured in RPMI 1640 medium (HyClone, USA) containing 10% fetal bovine serum (FBS, HyClone, USA), 100 U/ml penicillin, and 100 mg/ml streptomycin (Genom, China), at 37 °C with 5% CO2. 20 ng/ml IFN-γ (PeproTech, USA)- and TNF-α (PeproTech, USA)-treated cells to construct a cell inflammation model. siSTAT1, siEP300, and FAM-labeled siNC were purchased from Guangzhou RiboBio (Guangzhou, China) and transfected into cell line using Lipofectamine 2000 (lipo2000, Invitrogen, USA). Transfection efficiency of siRNA with lipo2000 was preliminarily assessed by a fluorescence microscope (Olympus U-RFL-T, Japan) 24 h after transfection, and the silencing effect of siRNA was further verified by qRT-PCR and WB experiments.

### RNA extraction and qRT-PCR

The total RNA of tissues and NCM460 cells was extracted by Trizol reagent (Invitrogen, USA) after treatment of TNF-α and IFN-γ for 24 h, and the reverse transcription was performed using TOYOBO ReverTra Ace kit (TOYOBO, Japan). mRNA expression was quantified using quantitative reverse transcription PCR (qRT-PCR) on Biorad CFX (Biorad, USA), and GAPDH was selected as a housekeeping gene. The primers were designed and synthesized by TSINGKE Biological Technology (Wuhan, China). The expression levels of mRNA were calculated using the comparative CT (2-ΔΔCT), and all experiments were performed with three biological replicates.

### Protein extraction and western blotting

Total protein in NCM460 was extracted after treatment of TNF-α and IFN-γ for 48 h. The protein in cells and colonic mucosa was extracted using RIPA lysis buffer (Boyotime, China) according to the reagent instructions. Western blotting was performed with the specific antibody, STAT1 (1:1000, Cell Signaling Technology, 14994S), p-STAT1 (1:1000, Cell Signaling Technology, 9167S), GAPDH (1:1000, Proteintech, 60004-1-Ig), EP300 (1:1000, Cell Signaling Technology, 4771 T), H3K27ac (1:1000, Cell Signaling Technology, 8173S), TNFAIP2 (1:500, Santa Cruz, sc28318), and LCP2 (1:1000, Santa Cruz, sc-13151).

### Mice feeding and tissue collection

The chronic mice colitis model was performed as previous [26]. In the acute colitis model, 8-week-old male mice were fed with water for 4 days following 3% DSS (MP Biomedicals) for one week. At the same time, in the inhibitor group, C646 (MCE, USA) was given by intraperitoneal injection at a concentration of 1 mg/kg, and the non-inhibitor group was given DMSO of the same concentration which was given every other day from the first day. At the same time, the control group kept drinking water until the three groups were killed at day 11. DAI was accessed based on weight loss, stool consistency, and the degree of intestinal bleeding [50]. 0.5 cm of the distal colon for all mice was used for hematoxylin–eosin (HE) staining.

### Protein preparation and coimmunoprecipitation

The extraction of protein was performed as previously described. Two microliters ProteinA/ProteinG Magnetic Beads (#19B002202, Beaver, China) were added to 200ul cell lysate and incubated for 45 min with gentle rotation at 4 °C. After centrifugation at 12,000 rpm/min for 1 min at 4 °C, the supernatant was equally split into two new 1.5-ml tubes. EP300 antibody (1:50, Cell Signaling Technology, 4771 T) and IgG were added, and the tubes were gently swirled and mixed overnight at 4 °C. After incubation overnight, 5ul ProteinA /ProteinG Magnetic Beads were added to each tube and mixed gently at 4 °C for 3 h. After centrifugation at 12,000 rpm/min at 4 °C for 1 min, the precipitate was washed with 500 μl 1 × wash buffer three times. Then, loading buffer was added to the precipitate and 15–30 μl of the sample was loading on SDS–PAGE for western blot.

### ChIP-PCR assay

We ground the tissue with a tissue grinder (Shanghai Jingxin Industrial Development Co., Ltd., China). Then, the tissue was fixed with 1% formaldehyde and incubated at room temperature for 10 min to make DNA protein cross-links. Then, glycine was added to stop the cross-linking and incubated at room temperature for 5 min. One milliliter tissue lysis containing protease inhibitors (MCE, USA) was added to suspend tissue, and then, tissue was sonicated using EPISONIC (USA) to get 200–300 bp of chromatin fragments. Immunoprecipitation was performed with STAT1 (1:1000, Cell Signaling Technology, 14994S), p-STAT1 (1:1000, Cell Signaling Technology, 9167S), and H3K27ac (1:50, ABclonal Technology, A7253). The chromatin DNA was extracted using DNA purification kit (TIANGEN, China), and the specific primers of TNFAIP2 and LCP2 enhancer were used for PCR. The primer sequences were as follows: TNFAIP2-F5′-GTGCCTTCCAGTCAGAGGAG-3′, R5′-GCATCATAGGGAGGTC.

AGGA-3′, LCP2-F5′-GGGGTTTGTGCAGAGAGAGA-3′, R5′-CTTTGCCCAGACCTACCAAG-3′.

#### Statistical analysis

All the experiments were performed in three times, and data were exhibited as mean ± SEM or mean ± SD. When the variance between the two groups was similar, Student’s t test was used to analyze data difference between two groups; if not the same, Welch’s t-test was used. Statistical analysis was performed using SPSS 17.0 software (IBM, USA), and GraphPad Prism 7.0 software (GraphPad software, USA). *p* < 0.05 was considered to be statistically significant.

## Data Availability

We have uploaded the data about RNA-Seq and ChIP-Seq to the GEO, RNA-Seq named GSE129454 and ChIP-Seq named GSE117062, respectively.

## Abbreviations

IBD: Inflammatory bowel disease
STAT1: Signal transducer and activator of transcription 1
EP300: E1A-binding protein p300
TFs: Transcription factors
LCP2: Lymphocyte cytosolic protein 2
TNFAIP2: TNF-α‐inducible protein 2
UC: Ulcerative colitis
CD: Crohn’s disease
HDAC: Inhibition of histone deacetylases
MDSCs: Myeloid-derived suppressor cells
LCA: Lithocholic acid
DCA: Deoxycholic acid
IFN-γ: Interferon γ
TNF-α: Tumor necrosis factor alpha
siRNA: Short interfering RNA
DSS: Dextran sulfate sodium
AOM: Azoxymethane
DAI: Disease activity index
GEO: Gene expression omnibus
